# Diffuse Large B-Cell Lymphoma Presenting as an Unusual Paraneoplastic Neurologic Syndrome Affecting the Central and Peripheral Nervous Systems

**DOI:** 10.7759/cureus.14590

**Published:** 2021-04-20

**Authors:** Anthony D Sorrentino, Amar H Kelkar, Nam H Dang

**Affiliations:** 1 Internal Medicine, Northwestern University Feinberg School of Medicine, Chicago, USA; 2 Oncology, University of Florida College of Medicine, Gainesville, USA

**Keywords:** paraneoplastic neurologic syndrome, peripheral neuropathy, diffuse large b-cell lymphoma, non-hodgkin lymphoma, chronic inflammatory demyelinating polyneuropathy

## Abstract

A 68-year-old man presented with a two-week history of ascending, symmetric, sensory neuropathy concerning an acute inflammatory demyelinating polyneuropathy that briefly responded to intravenous immunoglobulin (IVIg) therapy. The initial workup was negative for acquired causes. After three months of poor response to standard therapies, he was hospitalized for severe disability, unintentional weight loss, and additional, unexplained neurologic symptoms including cerebellar ataxia, dysarthria, and muscle twitching. Positron emission tomography revealed hypermetabolism isolated to the bone marrow. Bone marrow biopsy confirmed the diagnosis of diffuse large B-cell lymphoma (DLBCL). Due to rapidly worsening performance status, plasmapheresis was initiated prior to treatment with rituximab, cyclophosphamide, doxorubicin, vincristine, and prednisone (R-CHOP) chemotherapy. His symptoms initially improved following plasmapheresis and resolved with chemotherapy. One year following treatment, he remains in complete remission. This case describes a unique paraneoplastic neurologic syndrome involving the central and peripheral nervous system that responded well to plasmapheresis and systemic chemotherapy.

## Introduction

Paraneoplastic neurologic syndromes (PNS) are a heterogeneous group of immune-mediated, neurologic disorders associated with malignancy. Although some “classical” PNS exist such as Lambert-Eaton, subacute cerebellar degeneration, and opsoclonus-myoclonus, patients may present with a wide range of symptoms. We present a case of an unusual PNS associated with diffuse large B-cell lymphoma (DLBCL) masquerading as inflammatory demyelinating polyneuropathy. This case highlights the difficulty of diagnosing PNS, especially in cases of occult rather than overt malignancies. Treatment considerations are also discussed.

## Case presentation

**Figure 1:**
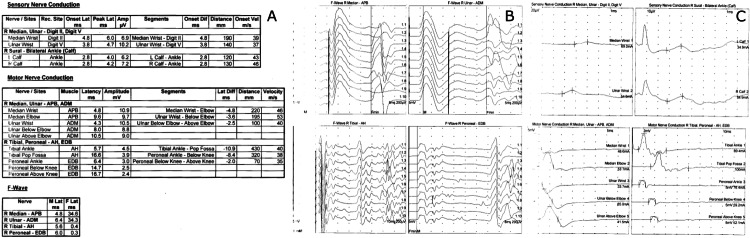
Electrodiagnostic studies.

A 68-year-old male presented to the ED at the local Veterans Administration Medical Center for severe lower extremity weakness and paresthesia. Three months prior, he first sought medical attention for progressive, ascending numbness and paresthesia of his lower extremities and hands. At that time, he met diagnostic criteria for acute inflammatory demyelinating polyneuropathy including characteristic electrodiagnostic and cerebrospinal fluid (CSF) analysis findings. He demonstrated moderate improvement with five doses of intravenous immunoglobulin (IVIg) at 0.4 mg/kg IV per day. One month later, he presented to the ED with worsening sensory neuropathy and weakness and new-onset dysarthria. Repeat electromyography/nerve conduction studies (EMG/NCS) showed progressive demyelination consistent with chronic inflammatory demyelinating polyneuropathy (CIDP) (Figure 1).

He was treated with an additional five doses of IVIg and oral steroids, this time with minimal improvement. In the subsequent weeks, he reported worsening lower extremity weakness, becoming too weak to stand, and the onset of several other neurologic complaints including dysarthria, mild dysphagia to thicker liquids and saliva, intermittent dizziness, and nocturnal leg twitching. He also exhibited constitutional symptoms including marked fatigue, unintentional weight loss of 25 pounds, and asthenia, associated with lower extremity muscle wasting. He had not experienced other classical B-symptoms, headaches, visual changes, abnormal bleeding or bruising, new skin rashes, myalgias, or neuropathic pain symptoms. He denied exposure to sick contacts, recent travel history, or tick bites.

Past medical history was significant for hypothyroidism, mild sensorineural hearing loss, and plaque psoriasis. He was up to date with age-appropriate cancer screening including colonoscopy and prostate-specific antigen (PSA) testing, which had both been normal. His only medication prior to the onset of his symptoms was levothyroxine 25 mcg daily per os. His past surgical history was only significant for bilateral cataract surgery. Prior to the onset of his neurologic symptoms, he was healthy and active, and was working as a scuba diving instructor. Over the course of three months, he had become progressively reliant on his wife for assistance with his activities of daily living. He denied any smoking history or illicit drug use including intravenous drugs and reported occasional social consumption of 1-2 glasses of wine on weekend nights but denied binge drinking. His family history included testicular mesothelioma in his father, but there was no other known history of cancer, bleeding disorders, thrombotic disorders, or neurologic disorders. By physical examination, the patient was cachectic with obvious signs of proximal muscle wasting. His vital signs were within normal limits. Examination of his cervical, axillary, and inguinal lymph nodes was unremarkable, and no rashes or bruising was noted. His abdomen was soft, without palpable masses or appreciable hepatosplenomegaly. Several abnormalities were noted in his neurologic exam, including left-eye horizontal nystagmus on left lateral gaze. His strength was grossly diminished, particularly in the lower extremities. Hyporeflexia was present, including 1+ patellar reflexes and absent upper extremity and ankle jerk reflexes. Sensory exam revealed hypersensitivity to pinprick and diminished sensation to soft touch and vibration in all four extremities. Finger-to-nose testing revealed dysmetria. Romberg’s test was positive. His gait was limited by his bilateral leg weakness but was consistent with ataxia with a spastic component.

**Figure 2:**
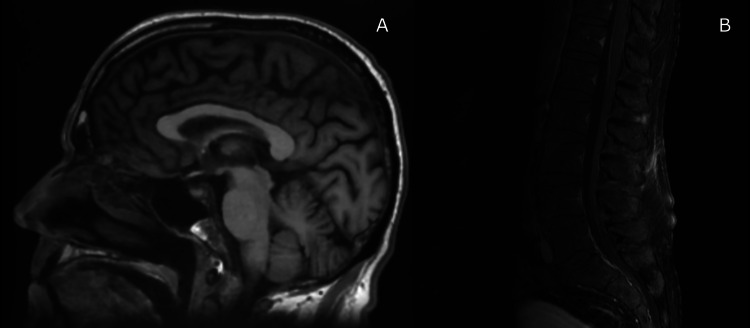
A) MRI of brain revealing normal parenchyma without masses. B) MRI of spine without cord compression or nerve root thickening. Questionable mild pial enhancement present.

Laboratory and imaging investigations performed during previous admissions included tests for associated infections (Lyme, HIV, hepatitis B and C, Epstein-Barr virus, cytomegalovirus, syphilis and bacterial cultures of the blood, urine, and CSF), vitamin and mineral deficiencies (vitamin B1, B6, or B12, folic acid, or copper), paraproteinemia (serum protein electrophoresis), inflammatory markers (C-reactive protein and erythrocyte sedimentation rate), and compressive neuropathy (MRI of the brain and spinal cord) and were all normal (Figure 2).

CSF analysis revealed a high CSF protein of 800 mg/L with a normal CSF white blood cell count of 5/mm3 and oligoclonal bands. EMG/NCS showed evidence of demyelination.

During the current admission, complete blood count was normal except for mild thrombocytopenia and anemia without iron deficiency. Serum electrolytes, coagulation studies, and hepatic and renal function tests were also largely within normal limits. An autoimmune paraneoplastic panel was negative for many common autoantibodies: ANNA-1 (Hu), ANNA-2 (Ri), PCCA-1 (Yo), amphiphysin, and CV2.1. CSF analysis was repeated with similar findings. CSF flow cytometry was negative for abnormal cells.

**Figure 3:**
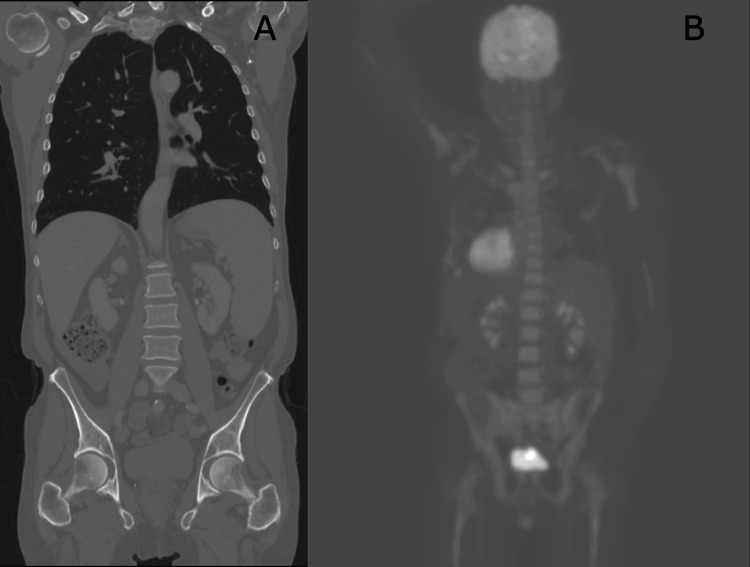
A) CT of chest, abdomen, and pelvis demonstrating splenomegaly and borderline hepatomegaly. B) Positron emission tomography demonstrating hypermetabolism in the axial and proximal appendicular skeleton.

Serum protein electrophoresis was performed with immunofixation, which revealed an elevated M-protein of 1.2 g/L with IgM kappa restriction. Positron emission tomography (PET) and CT imaging studies revealed splenomegaly with borderline hepatomegaly, and increased prevalence of mediastinal, axillary, and mesenteric lymph nodes without enlargement or increased fluorodeoxyglucose (FDG) avidity (Figure 3). 

**Figure 4:**
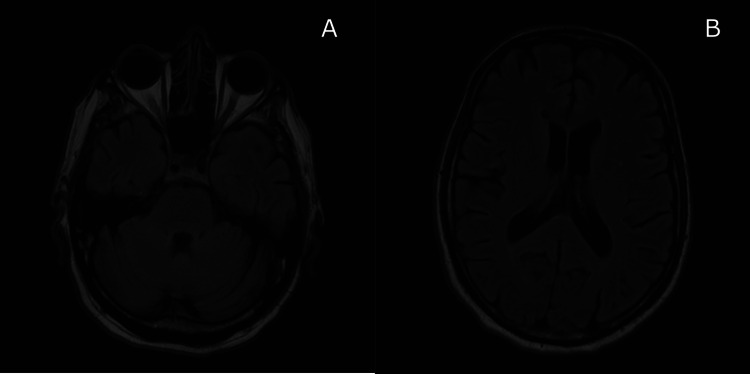
MRI of brain three months after the initial onset of symptoms revealed A) mild hyperintensity of both superior cerebellar peduncles on FLAIR imaging and B) a small punctate hyperintensity of the left inferior temporal white matter.

Moderate hypermetabolism throughout the bone marrow of the axial and appendicular skeleton was present. Repeat MRI of the brain showed FLAIR signal abnormality and slight contrast enhancement of the superior cerebellar peduncles bilaterally, more so on the right, suggestive of demyelination (Figure 4). 

Bone marrow biopsy revealed hypercellularity with DLBCL accounting for 15-30% of cells, and double-hit status was negative. Lactate dehydrogenase was 210 U/L and uric acid was 0.24 mmol/L. The patient’s international prognostic index (IPI) was calculated to be 3 (age >60, performance status >1, and stage IV disease), the intermediate risk for central nervous system (CNS) disease.

Treatment with multiagent chemotherapy including rituximab, cyclophosphamide, doxorubicin, vincristine, and prednisone (R-CHOP) was planned with the consideration of high-dose methotrexate to improve CNS penetration [1] due to the patient’s intermediate risk for CNS disease. Given the patient’s poor performance status, he was hospitalized for two cycles of plasmapheresis prior to initiation of chemotherapy. Following two cycles of plasmapheresis, the patient’s neurologic symptoms began to improve, and he had substantial improvement in performance status. Following cycle one of R-CHOP, the decision was made to not give high-dose methotrexate as the neurologic syndrome was felt to be paraneoplastic. Prolonged pancytopenia with severe neutropenia delayed treatment in the third, fifth, and sixth cycles, despite supportive care including scheduled granulocyte colony-stimulating factor. There were also multiple hospitalizations for febrile neutropenia during his treatment course. No dose reductions were made during treatment. Though eight cycles were offered, therapy was stopped after six cycles due to the patient's preference.

**Figure 5:**
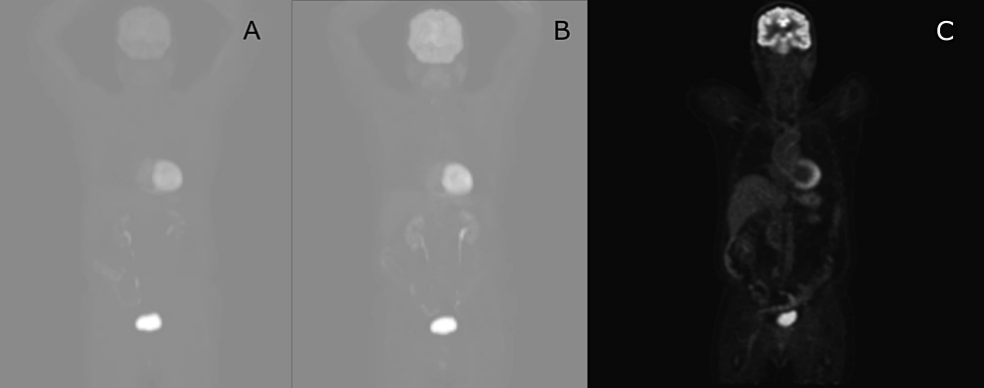
Positron emission tomography three, six, and 12 months after starting chemotherapy showing resolution of the hypermetabolism in the axial and appendicular skeleton. A: Three months; B: Six months; C: 12 months.

The patient has been seen at intervals of three, six, and 12 months since completion of therapy. Prior pancytopenia and hypogammaglobulinemia have fully resolved, and no new laboratory abnormalities were detected. PET/CT imaging at those intervals showed no evidence of active disease or splenomegaly (Figure 5).

**Figure 6:**
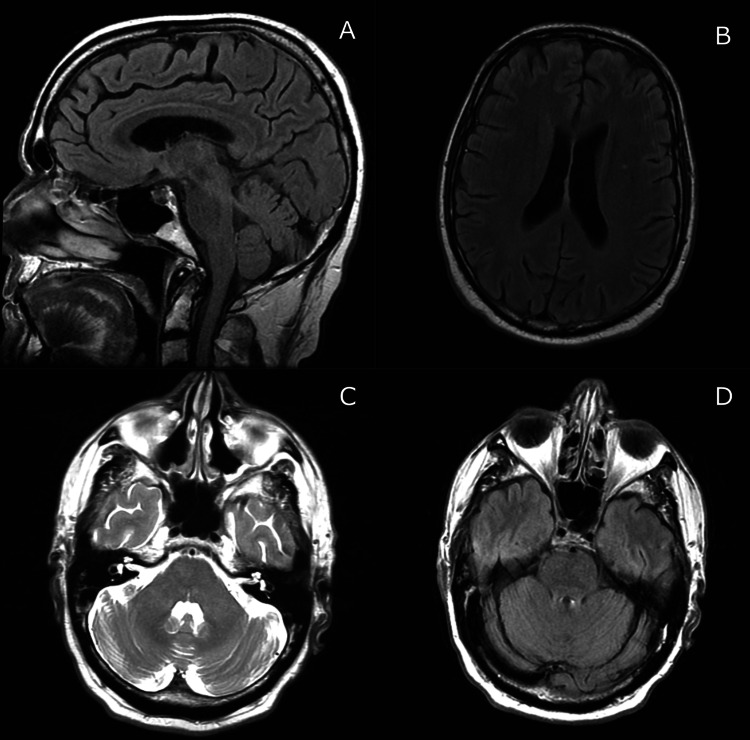
MRI of the brain 12 months after initiation of chemotherapy revealed no evidence of central nervous system lymphoma and resolution of the previously questioned cerebellar demyelination. A: Sagittal T1-weighted image; B: Coronal T1-weighted image; C: Coronal T2-weighted image; D: FLAIR image.

MRI brain was also repeated once at 12 months and did not demonstrate lymphoma or persistence of the possible cerebellar demyelination (Figure 6).

Due to the disease being primarily detected in the bone marrow, a biopsy was repeated at three and 12 months with low-normocellular marrow, and without evidence of lymphoma, dyspoiesis, or other clonal expansion.

He has not been hospitalized since completing chemotherapy. He has worked hard to regain strength, initially with physical therapy, followed by a daily weight-lifting and bicycling regimen at home. While he believes he has not fully regained his level of physical fitness from prior to therapy, he is highly active, and his only residual symptom is low-grade peripheral neuropathy of his bilateral feet. He recently resumed scuba diving instruction and deep-sea diving per his preference. We plan to follow the patient with repeat PET/CT imaging and bone marrow biopsy at two years, after which point he will be followed by labs and exams in conjunction with his primary care physician.

## Discussion

We present an unusual case of DLBCL presenting as a PNS affecting the central and peripheral nervous system that dramatically improved with plasmapheresis and R-CHOP chemotherapy. This case highlights the challenges of diagnosing PNS associated with occult malignancy. 

The patient was initially diagnosed with acute demyelinating polyradiculoneuropathy, also known as Guillain-Barré syndrome (GBS), and later CIDP after symptoms relapsed despite multiple cycles of steroids. Both are immune-mediated sensorimotor neuropathies. GBS is distinguished from CIDP by chronically progressive, stepwise, or recurrent symptoms typically lasting greater than two months. Many diagnostic criteria of CIDP have been proposed including the European Federation of Neurological Societies/Peripheral Nerve Society diagnostic criteria [2]. CIDP is primarily a clinical diagnosis. The diagnosis is supported by objective findings of cytoalbuminologic dissociation, improvement with immunomodulatory therapy, and findings of demyelination either from MRI, biopsy, or electrodiagnostics. It is, however, a diagnosis of exclusion. Therefore, initial workups of CIDP include age-appropriate cancer screening and screening for multiple myeloma. Further cancer workup may be pursued if certain risk factors, symptoms, or signs are present. 

In non-Hodgkin's lymphoma (NHL), findings of CIDP may precede other more specific signs of cancer, making diagnosis challenging. A systematic review of the literature from 1966 to October 2017 by Rajabally et al. found 54 cases of probable or definite CIDP in patients with hematologic malignancies [3]. This systematic review found that CIDP-like symptoms preceded the diagnosis of lymphoma in the majority of cases of NHL [3]. 

In our case, initial cancer screening was negative, including age-appropriate cancer screening and serum protein electrophoresis/urine protein electrophoresis (SPEP/UPEP) for multiple myeloma. When the patient presented with additional poorly localized neurological symptoms, weight loss, bicytopenia, and resistance to conventional CIDP treatments, a more extensive neoplastic workup was warranted. A PET/CT was performed revealing abnormal bone marrow metabolism without concerning masses. A bone marrow biopsy was required to make the diagnosis of NHL, namely DLBCL.

After diagnosing an NHL, the mechanism of neurologic involvement must be identified. Involvement can be either a result of direct invasion or immune-mediated. Direct invasion of the nervous system with cancer cells includes CNS lymphoma, leptomeningeal lymphomatosis, and neurolymphomatosis [4]. The annual incidence of primary CNS lymphoma in the United States is seven in 1,000,000 representing approximately 4% of CNS tumors. Secondary involvement occurs primarily in aggressive B-Cell lymphomas and is present in approximately 5% of cases [5]. Gadolinium-enhanced MRI is the imaging modality of choice for CNS involvement of NHL [6]. Leptomeningeal spread can occur with or without MRI evidence and is diagnosed with CSF flow cytometry, which has improved sensitivity to CSF cytologic analysis alone [7]. Neurolymphomatosis, tumor invasion of peripheral nerves, is highly fatal and is best detected on PET/CT [3]. 

Immune-mediated neurologic disorders include PNS, paraproteinemic neuropathies, and vasculitis [4,8,9]. Paraneoplastic neurological syndromes are a heterogeneous group of immune-mediated disorders affecting every level of the nervous system. Antigens on the tumor surface result in the production of onconeural antibodies that cross-react with neural tissue, which can lead to a wide range of presentations including several “classical” syndromes such as subacute cerebellar degeneration, Lambert-Eaton myasthenic syndrome, and limbic encephalitis. Over the last two decades, several of these onconeural antibodies have been discovered both aiding in the diagnosis and understanding of these disorders. 

A widely cited diagnostic criteria proposed by an international expert panel groups the spectrum of PNS based on the probability of diagnosis. Definite PNS includes patients with a “classical” syndrome and either an identified tumor or well-established onconeural antibody. On the other end of the spectrum, possible PNS includes patients with known cancer, an atypical syndrome, and no identified onconeural antibody [10]. 

The primary treatment of PNS is the treatment of the underlying malignancy. This reduces antibody production and associated immune-mediated inflammation. Immunomodulation with IVIg, plasmapheresis, corticosteroids or immunosuppressive agents such as cyclophosphamide are occasionally utilized as adjunctive therapy, but large clinical trials are lacking [11].

## Conclusions

This case highlights the difficulty in diagnosing occult malignancy when neurologic symptoms predominate. Ultimately, CIDP is a diagnosis of exclusion. Investigating for occult malignancies must be driven by pre-test probability of disease. In this case, at initial presentation, symptoms were classic for CIDP, and age-appropriate cancer screening and myeloma testing were negative. However, as the case evolved to include severe weight loss and fatigue, bicytopenia, and evidence of CNS involvement, a more thorough cancer investigation was demanded. 

Although several PNS associated with known malignancies have been well described and paraneoplastic panels are now available, our understanding of these syndromes is still incomplete. Plasmapheresis and IVIg may temporize antibody-mediated PNS especially in cases of extreme symptoms, but the mainstay of treatment remains to treat the underlying malignancy.
